# A qualitative study of HPV vaccine acceptability among health workers, teachers, parents, female pupils, and religious leaders in northwest Tanzania

**DOI:** 10.1016/j.vaccine.2012.06.025

**Published:** 2012-08-03

**Authors:** Pieter Remes, Veronica Selestine, John Changalucha, David A. Ross, Daniel Wight, Silvia de Sanjosé, Saidi Kapiga, Richard J. Hayes, Deborah Watson-Jones

**Affiliations:** a Mwanza Intervention Trials Unit, National Institute for Medical Research, P.O. Box 11936, Mwanza, Tanzania; b Social and Public Health Sciences Unit, Medical Research Council, 4 Lilybank Gardens, Glasgow G12 8RZ, United Kingdom; c National Institute for Medical Research, P.O. Box 1468, Mwanza, Tanzania; d London School of Hygiene and Tropical Medicine, Keppel Street, London WC1E 7HT, United Kingdom; e Unit of Infections and Cancer, Cancer Epidemiology Research Programme, Catalan Institute of Oncology, Av. Gran Via de l’Hospitalet 199-203, 08908 L’Hospitalet de Llobregat, Barcelona, Spain

**Keywords:** Human papillomavirus, Vaccine, Acceptability, Schools, Barriers, Tanzania

## Abstract

**Background:**

As human papillomavirus (HPV) vaccines become available in developing countries, acceptability studies can help to better understand potential barriers and facilitators of HPV vaccination and guide immunisation programs.

**Methods:**

Prior to a cluster-randomised phase IV trial of HPV vaccination delivery strategies in Mwanza Region, Tanzania, qualitative research was conducted to assess attitudes and knowledge about cervical cancer and HPV, and acceptability of and potential barriers to HPV vaccination of Tanzanian primary schoolgirls. Semi-structured interviews (*n* = 31) and group discussions (*n* = 12) were conducted with a total of 169 respondents (parents, female pupils, teachers, health workers and religious leaders).

**Results:**

While participants had heard of cancer in general, most respondents had no knowledge of cervical cancer, HPV, or HPV vaccines. Only health workers had heard of cervical cancer but very few knew its cause or had any awareness about HPV vaccines. After participants were provided with information about cervical cancer and HPV vaccination, the majority stated that they would support HPV vaccination of their daughter to protect them against cervical cancer. Opt-out consent for vaccination was considered acceptable. Most preferred age-based vaccination, saying this would target more girls before sexual debut than class-based vaccination. Potential side effects and infertility concerns were raised by 5/14 of participating male teachers.

**Discussion:**

Reported acceptability of HPV vaccination amongst parents, teachers and other community members was high in this population. Respondents stressed the need to provide adequate information about the vaccine to parents, that also addresses side effects and infertility concerns.

## Highlights

► The acceptability of HPV vaccination in Tanzania was studied qualitatively. ► Non-health workers knew nothing about HPV and HPV vaccines. ► Very few health workers (HWs) knew about HPV and HPV vaccines. ► After a brief explanation most of both groups supported HPV vaccination. ► Community and HW sensitisation will be needed before introducing HPV vaccination.

## 1 Introduction

Human papillomavirus (HPV) vaccines have the potential to significantly reduce the incidence of cervical cancer, the leading cause of cancer mortality among women in sub-Saharan Africa [1,2]. Two HPV vaccines have now been approved for use in many countries. These provide a high degree of protection against HPV 16/18 infections and associated cervical lesions [3–5]. The World Health Organisation recommends offering HPV vaccine to girls at ages 9–14, prior to sexual debut, since the vaccine has highest efficacy if girls have not already acquired HPV [6].

Many high-income countries and some middle-income countries have started national HPV vaccination programs, either school-based or on-demand programs, with vaccine coverage (completion of the 3-dose regimen) ranging from 9% (Greece) to 32% (US) and 76% (UK) [7–10]. In sub-Saharan Africa, two vaccine demonstration projects have been completed [11,12]; Rwanda has embarked on a national HPV vaccination programme [13,14], and Tanzania plans to start a similar programme in 2012. Research in Africa on HPV vaccine acceptability and delivery is needed to understand how best to deliver this vaccine to adolescent girls among populations who have little or no knowledge about cervical cancer, and may be suspicious of vaccines that target young women or a sexually transmitted infection (STI) [15–22].

Between August 2010 and June 2011, in preparation for a national HPV immunisation program, a phase IV cluster-randomised trial (NCT01173900) in schoolgirls in Mwanza Region, Tanzania, was conducted to measure the feasibility, uptake, and acceptability of two school-based HPV vaccine delivery strategies: age-based (all girls born in 1998) or class-based (all girls in Year 6 of primary school in 2010) [12]. We present findings from a qualitative sub-study conducted before the actual HPV vaccination started in August 2010. The sub-study's objectives were to learn what people knew about cervical cancer and HPV vaccination, whether they would find HPV vaccination acceptable, and how they viewed vaccine delivery and consent procedures. These findings were used to improve sensitisation and vaccination procedures within the trial and to assist preparations for a national HPV vaccination program.

## 2 Methods

The qualitative sub-study study took place in the two districts of Mwanza city and a neighbouring rural district (Misungwi), between March and August 2010. We conducted 31 in-depth interviews (IDI) and 12 group discussions (GD) with a total of 169 respondents, using a purposive sampling strategy with the purpose of recruiting particular kinds of respondents, in our case specific groups or types of people who would be involved in the different sites of school-based vaccination. Participants included parents/caregivers, female students, teachers, religious leaders (seven Christian and two Muslim), and health workers. Aside from parents in two group discussions (discussed below), these participants had not received any project-related sensitisation. A small monetary incentive (equivalent of 3 USD) was provided to adult participants to compensate them for the time spent during the interview or group discussion. For interviews with teachers, parents, and pupils, different school strata were selected: government urban, government rural, and private schools. When possible, individuals were recruited from the three strata (Table 1).

Head teachers assisted in recruiting parents, female students, and teachers; selection criteria were that these persons would be involved in the actual vaccination program, either as a parent, a student, or a teacher of Year 6 or 12-year-old girls. The girls selected were asked for written assent after their parents/caregivers gave their permission. Two group discussions were held with parents after a cultural dance and drama troupe performed a show on cervical cancer and HPV. We chose nine health facilities at random, representing rural and urban sites and interviewed one health worker in each, exploring the following themes: knowledge of cervical cancer and HPV, HPV vaccine acceptability, views on delivery strategies, decision-making, and other experiences with vaccines or school-based health services. When respondents demonstrated no knowledge of cervical cancer, HPV, and/or the HPV vaccine, the interviewer gave a brief, standard explanation about the planned HPV vaccination project, and then continued with questions. IDIs and GDs were recorded, transcribed and translated into English; the source and/or location of IDI and GD are given after quotations in the main results. Initial coding, which used a list of pre-set codes based on the research themes with further codes added that emerged during repeated readings, was reviewed by a second researcher who conducted the final analysis.

## 3 Results

### 3.1 Population characteristics

The age range of teachers and health workers interviewed was between 19–51 years and 33–55 years respectively. The 54 student respondents had a median age of 12 years and were aged between 11 and 17 years whilst parents were aged between 18 and 59 years. The majority of parents worked as farmers, fisherman or operate small businesses (e.g., food or vegetable sellers). Most had completed primary school; a minority (12/60) had completed secondary school. The religious leaders were aged between 35 and 50 years and came from the following denominations: Roman Catholic, Anglican, Lutheran, Tanzania Assemblies of God, African Inland Church, Seventh-Day Adventist, Glory City Church, and Muslim.

Large scale qualitative research suggests that the median age of sexual debut is approximately 14 although self-reporting in surveys suggests 16–17 years [23,24]. Primary school enrolment is generally very high in Tanzania: officially, the net attendance ratio for the primary-school age (7–13 years) population in Mwanza is 73.4% among boys and 76.3% among girls [25]. Part of the main trial preparations involved a check of pupil attendance records prior to the start of vaccination; the proportion of pupils absent on any one day ranged between 9.6 and 19.7% for Year 6 pupils and between 8.1 and 23.5% for all pupils in Years 4–7 [12].

### 3.2 Knowledge of cervical cancer, HPV, and HPV vaccine

Nine female health workers were interviewed; all but one had two years of nursing education. All had heard of cervical cancer but their knowledge was limited and often inaccurate. When asked about cervical cancer symptoms, they mentioned vaginal bleeding, smelly vaginal discharge, or pain during sexual intercourse. Only two nurses identified HPV as the cause of cervical cancer. Both had heard about HPV through preparatory work for an immunogenicity and safety trial of the bivalent HPV vaccine in Mwanza (2009–2010). Another nurse had heard of HPV vaccines on the radio but could not remember any details. All nurses mentioned a wide range of, sometimes incorrect, causes of cervical cancer such as poor genital hygiene, early age at childbirth, frequent childbirth, abortion, wearing nylon undershorts and insertion of traditional medicines.

Most parents recognized cancer as a serious, potentially deadly illness, but knew little about cervical cancer. Two parents (participating in an GD) had heard about it on the radio but did not remember any details. One 53-year-old father (participating in an IDI) heard information on the radio but incorrectly thought that cervical cancer affected women during pregnancy or menstruation, when poor vaginal cleansing caused women to contract germs and then cancer. Four parents (GD) and two mothers (IDI) had heard of uterine, but not cervical cancer. No parent had heard about HPV or the HPV vaccine. The female pupils had heard of cancer in general, but none of the 49 girls in GDs had heard about cervical cancer, HPV or the HPV vaccine. Similarly, teachers had heard of cancer but only 1 of 37 knew about cervical cancer, and no teacher had heard of HPV or the HPV vaccine. One 48-year-old female teacher (IDI) talked about a family member who had “died of cervical cancer” but recalled little about the disease. Religious leaders interviewed knew about cancer in general but nothing of cervical cancer, HPV, or the HPV vaccine.

### 3.3 Attitudes towards HPV vaccination

Most respondents welcomed a vaccine that prevents cervical cancer. Almost all the adults said they would allow their daughter to be vaccinated since “prevention is better than cure” (female teacher, GD Malulu). All the girls interviewed said they would like be vaccinated to avoid a dangerous disease like cervical cancer.

Five (35%) of the 14 male teachers participating in this study (GD Malulu) dissented and said they would not allow their own daughters to be vaccinated. They feared side effects; especially whether the vaccine would have a potential effect on future reproduction: “vaccinations in this country that are linked to issues of reproduction have had very bad results later on,” or the vaccine could “disorder and destroy the eggs that a girl has, and reproducing would be a problem.” The aunt of one student was suspicious of the vaccine and had told her: “they are coming to implant cancer in people… they are coming to reduce reproduction” (GD Nyakato).

Most participants trusted the safety of the vaccine, since it had been explained that the Tanzanian government had approved the vaccine: “I know the government cannot do something malicious to children” (parent, GD Mirongo).

All parents stated they would agree to have their daughters vaccinated, but some hesitated when confronted with an unknown infection (HPV), disease (cervical cancer), and vaccine: “That disease you are talking about, we are completely in the dark about it” (parent, GD Mkolani), and “The vaccine will have a benefit if it does not have harmful side-effects” (parent, GD Mirongo).

The five male teachers (GD, Malulu) who opposed vaccination also commented that the vaccine might give girls a license to start sexual activity: “if this is introduced, a person would have the freedom to do anything.” A few religious representatives also echoed this concern but most found the vaccine a ‘good thing’ because it would protect adolescent girls. No parents thought that the vaccine would encourage sexual activity among the targeted girls.

Generally, teachers, parents, students, and health workers preferred age-based vaccination as they believed that this would target more students who had not yet started sexual activity; choosing students in School Year 6 [where the mean age is 13.9 years (range 11–22 years)] would include a greater age-range and older girls who might have started sex. Participants suggested vaccinating much younger girls: “a ten-year-old child has already started with sex, the ones who have not started are those aged seven” (parent, GD Mirongo). A few suggested testing girls’ HPV status before vaccination. If class-based delivery was to be used, participants preferred classes lower than Year 6. A few parents preferred class-based delivery because of simpler logistics, since each girl's age would not need to be checked. Other interviewees focused more on student understanding and preferred 12-year-olds: these would be “mature enough” to understand the vaccination information and could help to “educate parents” (teacher, GD Serengeti); those in Year 6 would “value” the vaccine more (health worker, IDI Makongoro).

A few girls and a few parents said that boys might also want the vaccine, and could feel discriminated against or jealous of the girls, but the general consensus was that, because cervical cancer only affects girls, boys would understand if only girls were vaccinated. Health workers anticipated that questions from boys could be resolved by explaining the underlying reasons: “when we educated [the boys], they understood” (health worker, IDI Nyakato).

A few respondents asked how out-of-school girls could get the HPV vaccine. Some parents suggested organising door-to-door visits to identify and vaccinate all girls of a certain age, regardless of education status. Some religious representatives asked what could be offered to their wives and adult sisters.

### 3.4 Previous experiences with school-based/health-centre vaccinations

The majority of participants were positive about other vaccinations, such as for measles, tetanus or polio. They saw that “when children are vaccinated, they grow up healthy and do not get that disease” (parent, GD Kayenze). Health workers confirmed that there was “much awareness” about infant vaccinations; mothers knew that minor side-effects (a fever, soreness) might occur post-vaccination (IDI Igoma).

Reactions to a new HPV vaccine being delivered through primary schools were influenced by past experiences with vaccinations and/or school-based health programs. Many participants remembered rumours undermining previous vaccination or de-worming campaigns [26–28], and stressed the importance of adequate information about the new vaccine to reduce the likelihood of rumours undermining future programmes.

### 3.5 Health workers’ views on adding HPV vaccination to health services

When asked about adding HPV vaccination to their workload, health workers all mentioned familiar concerns about public health services: insufficient staff serving a large population and lack of transport. One nurse said, “some places are far away and some of us have become old” and, with not enough staff, “you might find yourself alone at work for the whole month” (IDI Nyegezi). Health workers encountered various shortages; of drugs, vaccines, or consumables: “we might lack drugs for two weeks… sometimes we have the drugs but would not have the syringes” (IDI Makongoro).

One nurse summed up ways to alleviate these issues: the necessary “facilities” for storing vaccine, “enough medicines,” “motivation [i.e. salary supplements] for those who go to do the work,” and training “so that she can administer the vaccine correctly” (IDI Igoma).

### 3.6 Views on sensitisation and opt-out consent

All respondents emphasised that parents need appropriate information and intensive sensitisation about HPV infection and the new vaccine. Without this, parents would quickly oppose a new vaccine: “we’d charge you [in court]” (parents, GD Mirongo). All viewed school-based meetings as an essential sensitisation strategy: “[parents] should get educated like how you [the interviewer] have come here” (parents, GD Usagara). Teachers said inviting parents to school meetings was not always successful. Not all parents may attend and, even when they did, “you might educate the wife, but when she gets home to her husband, he refuses” (health worker, IDI Sangabuye). Some suggested adding radio and television announcements, so that even those who do not attend meetings are informed.

Students thought sending letters to parents via students would work, provided they themselves also received sufficient information: “It won’t be difficult [to deliver letters] because many children will agree to be vaccinated and very few won’t want to get the vaccine.” (IDI Buhongwa). Most respondents liked the letter strategy but some teachers cautioned about relying on written information: not all parents know how to read.

Most teachers, parents, and students said it was necessary to get parental permission, but not necessary to ask each parent for individual written consent. Most interpreted consent as a process whereby parents would be informed about the school-based vaccination programme, either by letters, meetings, by the targeted child, or other types of announcements (like radio or television); parents could refuse to allow their child to be vaccinated by making this known to the school or by keeping the child home on vaccination day. A few teachers (GD Ng’ombe) suggested that active consent should be required from parents, or that parents should accompany their daughter on the day of vaccination to ensure that parental wishes are respected. Teachers feared parents might threaten them at school, as happened during past health programmes, or take them to court. Some health workers suggested that teachers might have coerced their students during prior vaccination campaigns: “when we go to administer a vaccine, we find the teachers have gathered the girls, and they are standing by the door with a stick, …” (health worker, IDI Pasiansi).

Some parents, teachers and students said that if a student has sufficient understanding and wants to be vaccinated, she should get the HPV vaccine even if her parent(s) refused. “The child ought to be given the vaccine because it's for her benefit, provided she's willing and has got sufficient education. If the parent isn’t willing, it's the right of the child to get it” (teachers GD Serengeti); “I should be vaccinated because I’m the one who’ll contract the disease” (student, IDI Nyamhongolo).

Health workers were accustomed to giving infant and child vaccinations without parental consent. With nationally-mandated vaccinations, health workers go to schools, inform the teachers, and on vaccination day, inform and vaccinate the children. These are vaccines that “the community knows and understands [to not be] harmful” (health worker, IDI Igoma). Most health workers felt that, if the government mandates HPV vaccine as part of the school vaccination program and the community has been ‘educated’, this should be sufficient. Two (of nine) health workers said children should not be vaccinated if their parents refuse, but health workers should try to convince these parents of the vaccine's benefit. Most health workers said that if the child understands and wants the vaccine, she should be vaccinated: “what I aim at is to save the life of the child, not the parent” (IDI Nyegezi). Another said: “A child is yours when she's in the womb. Once she's born, she belongs to the government … it can protect her” (IDI Butimba).

## 4 Discussion

We found that teachers, parents, pupils and health workers interviewed in our qualitative sub-study had limited or no knowledge about cervical cancer, HPV, and the HPV vaccine. Generally, most welcomed a vaccine to prevent cervical cancer and most parents said they would agree to have their daughter vaccinated although some adopted a “wait and see” approach. Most had a strong belief that vaccines prevent diseases. Our findings are similar to formative research results by PATH in Uganda, Peru, Vietnam and India prior to HPV vaccination [29,30], and recent studies on vaccine acceptability in Ghana, Botswana, Kenya, and South Africa [31–34]. In a study amongst 147 Kenyan women seeking health services there was little knowledge about either cervical cancer or the HPV vaccine [31]. Findings were similar in South African antenatal attenders [34]. In Botswana, awareness of cervical cancer was higher amongst many adults (mostly female) but again, few had heard of HPV vaccine [32]. In a Ghanaian study among 264 women, ages 18–65, where most had received higher education after secondary school, 87% of study participants had heard about cervical cancer and 40% about the HPV vaccine [33]. Despite variability in cancer and vaccine awareness, in all of these sub-Saharan studies, the majority of the women were willing to vaccinate their child.

Anti-fertility rumours, raised as a potential issue for the vaccine in our study and the study in Uganda, are widespread in Africa in relation to vaccines and health-related products and reflect underlying suspicions about public health interventions [35,36]. People may object to imported, foreign drugs and new medical interventions; knowing that the HPV vaccine has already been administered in Africa and is approved by the Tanzanian government was thought to be persuasive by many respondents.

Issues of power and control over health emerged in the discussions about opt-out consent. Health workers saw public health actions as mandatory and considered that individual parent consent was not a necessary part of national immunisation policy, although provision of information to parents and communities was important. This was also stressed by other respondents. In Mwanza, parents wanted to be involved in the decision-making process but the consensus was that opt-out consent was acceptable, and there was considerable support for a girl's right to be vaccinated, even if parents refused their consent. Uganda's pilot HPV vaccination program also used a similar opt-out approach [20]. No parents in our study reported concerns that the vaccine might stimulate sexual activity, a concern that has sometimes emerged in high-income countries [37,38].

Many respondents among the different groups (teachers, parents, health workers, religious representatives, female pupils) stated that parents should be given adequate information about the vaccine and cervical cancer, and that a school meeting would be an essential strategy to reach the parents. People were eager to learn about the HPV vaccine. Religious leaders reported that this was the first time that staff from a health programme had come to discuss a health intervention with them, and that they would discuss cervical cancer and HPV vaccination with their congregations.

Limitations of the qualitative sub-study included the fairly small purposive samples and the fact that, in schools, a teacher selected the parent, student and teacher participants for GDs who might have been the most accepting of new health interventions. However, the interviewer then selected IDI participants from the groups. These included several teachers who opposed vaccination, parents who asked critical questions, and female students who stated they would defy parental wishes in terms of accepting vaccine.

In USA, beliefs about the safety of vaccines, likelihood of HPV infection, as well as doctor's recommendations, have been associated with increased HPV vaccine acceptability [39–41]. In Mwanza, anti-fertility rumours, experience of previous school-based health interventions for girls, and lack of knowledge about cervical cancer in targeted communities, including amongst health workers, could be a potential challenge to vaccine uptake. It will therefore be essential that correct information about HPV vaccination is provided to parents, pupils, community members and key personnel (teachers, health workers) to help prevent the emergence and/or spread of rumours before and during HPV vaccination programmes.

In light of the recent price reduction of the Gardasil^®^ vaccine for low-income countries [42], many African governments may now consider adding the HPV vaccine to their national programs. Our research identified key issues related to vaccine acceptability and allowed adaptation of communication materials for the subsequent HPV vaccination demonstration project in Mwanza. Our findings also informed health worker training on issues related to obtaining parental agreement to vaccinate daughters, and rumour management. For a successful national programme on cervical cancer prevention, health workers should acquire additional training on the disease and prevention strategies. Adequate sensitisation, through school and/or community meetings and mass media, of all relevant populations, including parents, students, teachers, community and religious leaders will be essential for the success of a national HPV vaccination campaign in Tanzania.

## Figures and Tables

**Table 1:** Number of group discussions and in-depth interviews.

Research method	Teachers	Parents	Female students	Health workers	Religious leaders
Group discussions	3 (mixed sex) (*n* = 21F, 14M)	5 (mixed sex) (*n* = 30F, 24M)	4 (*n* = 49F)	–	–
In-depth interviews	1F, 1M	4F, 2M	5F	9F	2 Muslim 7 Christian

F = Female; M = Male.
